# Nutritional Assessment and Antioxidant Activities of Different Varieties of *Vigna radiata*

**DOI:** 10.1155/2014/871753

**Published:** 2014-10-15

**Authors:** Riaz Ullah, Zain Ullah, Salem S. Al-Deyab, Muhammad Adnan, Akash Tariq

**Affiliations:** ^1^Department of Chemistry, Government College Ara Khel, Frontier Region Kohat 26000, Pakistan; ^2^Department of Chemistry, Gomal University, Dera Ismail Khan, Khyber Pakhtunkhwa 29050, Pakistan; ^3^Petrochemical Research Chair, Chemistry Department, College of Science, King Saud University, P.O. Box 2455, Riyadh 11451, Saudi Arabia; ^4^Department of Botany, Kohat University of Science and Technology, Kohat 26000, Pakistan

## Abstract

Three cultivars of *Vigna radiata*, namely, NM-92, NM-98, and NM-06, were analyzed for their proximate composition. The samples were also tested by HPLC for amino acid content. The data showed that all the varieties had same moisture level. The maximum ash content (4.29%) was present in NM-92, and crude fat (2.26%) was highest in NM-98 while NM-06 contained maximum amount of crude protein. About eighteen types of amino acids were detected in each of the three varieties. Acidic amino acids, that is, aspartic and glutamic acids, were in considerable amount ranged from 13 to 23% followed by leucine, isoleucine, alanine, valine, lysine, phenyl alanine, serine, and arginine which fell in the range of 3–8% of total protein. The maximum amount (13.00 and 22.80%) of aspartic and glutamic acids was present in NM-98. Similarly arginine (6.83%) and serine (5.45%) were also in highest amount in this variety. Leucine (7.46%) was maximum in NM-92 variety. NM-06 contained almost all the amino acids in lesser quantity except for few like threonine, proline, glycine, and alanine. It was concluded from the present study that varieties were of different nutritional value and HPLC was a sensitive method for amino acids determination. Antioxidant activities of all three varieties were also assayed and showed significant results.

## 1. Introduction

Legumes are those species of family Leguminosae (Fabaceae) which are consumed directly by human beings, most commonly as mature dry seeds but occasionally as immature seeds enclosed in pods [1]. The leguminous grains, also known as pulses, are famous for their high quality protein content. Grain legumes are often considered as meat substitutes for people in less developed countries [2]. One member of legumes known as Mung bean (*Vigna radiata*), also called “green gram,” mainly grown in tropical regions of Asia, is an important summer-growing, annual crop [3].* Vigna radiata* has a short growing season and is relatively drought-tolerant. Mung bean is thought to be originated in South and Southeast Asian regions. It is widely grown in India, Pakistan, Bangladesh, Myanmar, Thailand, Philippines, China, and Indonesia. It is the second major pulse crop in Pakistan which has covered an area of 200 thousand acres with annual production of about 93 thousand tones equal to 446 kg per ha [4]. In KPK various regions like Malakand, Kohat, Karak, Bannu, and Dera Ismail Khan districts are famous for its production; however total production in the province is 12.6 tones on 8.1 hectors, so we selected different varieties of Mung bean from different regions of Khyber Pakhtunkhwa (KPK), Pakistan [3, 5].* Vigna radiata* fits well in our cropping system and it is grown as intercrop with other Kharif crops [6]. It contains isoflavoineds having estrogen and antioxidant activities, used as prevention of many diseases such as cancer; it also exhibits antimicrobial and insecticidal activities [7]. There are two main varieties, golden and green, the name denoting the color of dry seed. The climate and soil requirements of* Vigna radiata* are sandy loam soil with moderate moisture content. Mung bean is resistant to most of the diseases and insects pests of legume family.* Vigna radiata* is used as supplement for cereal based human diets due to its high lysine content. The grain has about 19.05 to 23.86% protein, 247.67 to 277.3 mg/100 g calcium, and 5.03 to 12.63 mg/100 g iron [8]. The nutritional quality of these grains may be impaired due to the presence of trypsin and other such antinutrients. This may cause low protein digestibility and minerals availability in legumes. In rural areas the immature green pods are also used as vegetables.* Vigna radiata* stalks, leaves, and husks constitute a significant proportion of livestock feed. The sprouted beans are consumed as cooked vegetable, usually in oriental dishes, or eaten raw in salads [9]. Protein of* Vigna radiata* is known as complete protein due to the presence of many of essential amino acids. Amino acids are required by man and other animals for their normal growth and health. Every cell of the body contains thousands of different proteins, each digested to carry specific function. And each protein is composed of a basic set of 20 amino acids. The acidic part (carboxylic group) transfers a proton to the basic part amino group (NH) that results in zwitterion. Amino acid in the zwitterion form is electrically neutral because the negative charge is balanced by the positive charge [10].

In the developing countries, like Pakistan, India, and Bangladesh, where mostly people are vegetarian for economic and religious reasons, they usually suffer from protein malnutrition problems as their diet is lacking lysine, so they are requested to eat food legumes because they are rich in lysine [11]. Keeping in view the importance of legumes, this work was an endeavor to highlight the nutritional importance by analyzing* Vigna radiata* for its certain chemical constituents, that is, proximate composition and amino acid profile.

## 2. Material and Methods

### 2.1. Sample Collection

Three varieties of* Vigna radiata,* that is, NM-92, NM-98, and NM-06, were collected from National Institute for Food and Agriculture (NIFA) Research Centre, TARNAB, Peshawar. All the samples were collected in one-kilogram plastic bags and were stored at room temperature. Similarly all the three verities were crushed and dipped in methanol for one month. It was shaken throughout and finally methanol was evaporated through rotary evaporator. The crude extracts of methanol of all three varieties were studied for antioxidant activities.

### 2.2. Sample Preparation

Seeds of each variety were shifted from dirt and malformed kernels and then samples were stored at room temperature and one-third of the seeds were ground in the grinder and then further ground to fine particles by chopper in the laboratory for chemical analysis.

### 2.3. Proximate Composition

Samples of* Vigna radiata* varieties were analyzed for crude protein, crude fat, ash, and moisture by standard methods of AOAC [12].

### 2.4. Moisture Contents

Moisture was determined by oven drying method. 1 g of each sample was accurately weighed in a Petri dish. The partially covered Petri dish was placed in an oven at 105°C for 6 hours. After cooling the Petri dish in the desiccators for 30 minutes, it was reweighed. The percent moisture was calculated as

(1) $$\begin{array}{c} \text{Moisture}\;\text{content}\left(\%\right)=\frac{\text{Weight}\;\text{loss}\;\mathrm{on}\;\text{drying}\times 100}{\text{weight}\;\text{of}\;\text{sample}}. \end{array}$$

### 2.5. Crude Fats

Crude fat was determined by ether extract method using Soxhlet apparatus. 1 g of each moisture sample was wrapped in filter paper, placed in thimble, and then introduced into extraction tube. Receiving flasks were filled up to 1/3 with hexane and fitted into apparatus. After complete extraction, the hexane from the receiving flasks was evaporated on water bath, the ether extract was dried in an electric oven, and the flasks were reweighted. The percent crude fat was determined by using the following formula:

(2) $$\begin{array}{c} \%\text{Crude}\;\text{Fat}=\frac{\text{Weight}\;\text{of}\;\text{ether}\;\text{extract}\times 100}{\text{weight}\;\text{of}\;\text{sample}}. \end{array}$$

### 2.6. Crude Proteins

One gram of each sample was taken in digestion flasks. 8 g of digestion mixture, that is, K_2_So_4_·CuSo_4_ (8 : 1), and 12 mL of concentrated H_2_So_4_ were added. Some pieces of fumic stones were also added to avoid bumping of the solution. Digestion was carried out by heating the mixture until it becomes clear.

After cooling, the digest was transferred to 100 mL volumetric flask and volume was made up to the mark with distilled water. Distillation was carried out in Markham Still Distillation apparatus. 10 mL of digest was introduced in the distillation tube through funnel. Then 10 mL of 40% NaOH was gradually added through the same way. Distillation was carried out for at least 10 minutes and NH_3_ produced was collected as NH_4_OH in a conical flask containing 20 mL of 4% boric acid solution with few drops of modified methyl red indicator and during distillation yellowish color appears due to ammonium hydroxide.

The distillate was titrated against standard 0.1 N HCL solutions till the appearance of pink color. A bland (experiment without sample) was also run through all the steps as above. Percent of crude proteins was calculated by multiplying the percent nitrogen with appropriate factor:

(3) $$\begin{array}{c} \frac{\%\text{N}=\left(S-B\right)\times N\times 0.014\times D\times 100}{\text{Weight}\;\text{of}\;\text{sample}\times V}, \end{array}$$

where *S* = sample titration reading, *B* = blank titration reading, *N* = normality of HCL, *D* = dilution of sample after digestion, *V* = volume taken for titration, and 0.014 = Milliequivalent Wt. of Nitrogen Crude proteins (%) = %N × 6.25.

### 2.7. Analysis of Amino Acid by HPLC

Six test tubes (1.5 × 10 cm) were washed and placed in oven at 100°C for 20 minutes for drying. Each dried test tube was put 40 mg dried ground Mung bean sample, 10 mL 6 N HCL and small portion of phenol was added. All the tubes were placed in vacuum desiccators and 700 mm Hg vacuum was created in order to stop the breakdown of amino acids by oxidation reaction. Desiccators were then placed in oven at 100°C for three days. 25 microliters of each hydrolysate was transferred to sample tube (6 × 50 mm) and was dried in vacuum and 50 microliters of dissolving reagent (methanol + distilled water + TEA; 2 : 2 : 1) was added to sample tube, mixed, and dried twice in vacuum. After this 50 derivatizing reagents containing methanol, TEA, water, and PITC (7 : 1 : 1 : 1) were added and incubated at room temperature for 20 minutes followed by drying in vacuum for 10 minutes. Methanol was then added to each sample tube and dried twice in vacuum. The residues were then dissolved in 200 microliters of solution containing disodium hydrogen phosphate and acetonitrile (95 : 5); 20 microliters was injected in HPLC and the data was recorded by SW 32 software system.

### 2.8. Antioxidant Bioassay

DPPH (diphenylpicrylhydrazyl) method was adopted for antioxidant activities. Test samples were allowed to react with stable free radical, 1, 1-diphenyl-2-picrylhydrazyl radical (DPPH) (from Sigma Aldrich), for half an hour at 37°C. The concentration of DPPH was kept as 300 *μ*M. The test samples methanol extracts of NM-92, NM-98, and NM-06 were dissolved in DMSO while the DPPH solution was prepared in ethanol. After incubation, decrease in absorption was measured at 515 nm using multiplate reader (Spectra MAX-340). Percent radical scavenging activity by samples was determined in comparison with a DMSO treated control group [12]. % radical scavenging activity was calculated by using the following formula: %RSA = 100 − {(OD  test  compound/OD  control)} × 100  [13].

## 3. Results and Discussion

Three varieties of* Vigna radiata* (NM-98 and NM-06) obtained from NIFA (Nuclear Institute of Food and Agriculture) were analyzed for their important proximate composition and amino acid contact. The data was presented in the form of table and graphs as shown below.

The data (Table 1) showed that all the varieties had same moisture level. The maximum ash contact (4.29%) was present in NM-92. Crude fats (2.26%) were highest in NM-06 which contained maximum amount of crude protein. The present data was in good line with [14] while studying the effect of processing on different pulses containing Mung beans. The reported ash content was up to 3.34%, the crude fat was up to 1.34%, and the crude protein was up to 22.90%. The data was also in good comparison with [1, 3] also reporting same kind of data while analyzing different Mung bean cultivars.

About eighteen amino acids were detected (Table 2) in each of the three varieties. The data showed that acidic amino acids, that is, aspartic and glutamic acids, were in considerable amount ranging from 13 to 23%, followed by leucine, isoleucine, alanine, valine, lysine, phenyl, alanine, serine, and arginine which fall in the range of 3–8% of total protein. The maximum amount (13.00 and 22.80%) of aspartic and glutamic acids was present in NM-98. Similarly arginine (6.38%) and serine (5.45%) were also in highest amount in this variety. Leucine (7.46%) was in NM-92 variety. NM-06 contained almost all the amino acids in lesser quantity except for few like threonine, proline, glycine, and alanine. The present data was in good line with [15] which studied the nutritional composition and antinutritional factors of* Vigna radiata* seeds as affected by home traditional processes. The data was also close to the results of [16] which analyzed various* Vigna radiata* cultivars for their nutrition.

Mung bean (*Vigna radiata*) belongs to family Leguminosae (Fabaceae) and is the second major pulse crop in Pakistan. It is used as a supplement for cereal based human diets due to its high lysine content. The grain has about 19.05–23.86% protein, 247.67–277.3 mg/100 gms calcium, and 5.30 to 12.63 mg/100 gms iron. The nutritional quality of these grains may be impaired due to the presence of trypsin and other such antinutrients. In the rural areas the immature green pods are also used as vegetables. Mung bean stalks, leaves, and husks constitute a significant proportion of livestock feed. The sprouted beans are consumed as cooked vegetables, usually in oriental dishes and are eaten raw in salads. Three cultivars of Mung beans, NM-92 and NM-98, were analyzed for their proximate composition. The samples were also tested by HPLC for amino acid content. The data showed that all the varieties had same moisture level. The maximum ashes content (4.29%) was present in MN-92. Crude fats (2.26%)  were highest in MN-98 while MN-06 contained maximum amount of crude proteins. About eighteen types of amino acids were detected in each of the three varieties. Acidic amino acids, that is, aspartic and glutamic acid, were in considerable amount ranged from 13 to 23% followed by leucine, isoleucine aniline, valine, lysine phenyl alanine, serine, and arginine which fell in the range of 3–8% of total protein; the maximum amount (13.00 and 22.80%) of aspartic and glutamic acid is present in MN-98. Similarly arginine (6.83%) and serine (5.45%) were also in highest amount in this variety. Leucine (7.46%) was maximum in MN-92 variety. MN-06 contained almost all the amino acids in lesser quantity except for few like threonine, praline, glycine, and alanine.

It was concluded from the present study that different varieties have different nutritional values and HPLC was a sensitive method for amino acid determination.

### 3.1. Antioxidant Activities

Free radicals are responsible for many diseases like cancer and AIDS. Antioxidants due to their scavenging activity are useful for the management of those diseases. DPPH stable free radical method is a sensitive way to determine the antioxidant activity of plant extracts [13]. Keeping in mind the importance of antioxidant activity, the methanolic crude extracts of all the three varieties of* Vigna radiata*, namely, NM-92, NM-98, and NM-06, were screened for antioxidant activity. Results obtained are given in Table 3. From Table 3, it is clear that NM-92 showed the highest activity 66.08%.±0.01 followed by NM-98, showing 64.15%.±0.01. It is comparable with standard showing result of 95.12% ± 0.01.

## 4. Conclusion

It was concluded that* Vigna radiata* contained various nutrients in appreciable quantities. All the cultivars contained crude protein in maximum amount which were comparable to those of animals sources. The HPLC method was used for amino acids determination was accurate regularly be utilized for the same purpose. It also showed significant antioxidant activities. It is recommended that the* Vigna radiata* due to high nutritional value and antioxidant potential could be used in daily human diet which would be helpful in fulfilling the protein need of the body. It is also recommended that utmost precautions should be taken while hydrolyzing samples for HPLC recommendation of amino acid, because high risks of oxidation contamination with other proteins are present.

## Figures and Tables

**Table 1:** Proximate composition of *Vigna radiata* varieties.

Parameters %	Varieties		
	NM-92	NM-98	NM-06
Moisture	4.05	4.04	4.00
Ash	4.29	3.51	3.93
Crude fat	1.15	2.26	1.68
Crude protein	13.20	14.77	17.05

**Table 2:** Amino acid profile (g/100 g of protein) of *Vigna radiata* varieties.

Amino acids	Varieties		
	NM-92	NM-98	NM-06
Tyrosine (C_9_H_11_NO_3_)	2.57	2.77	2.37
Phenyl alanine (C_9_H_11_NO_2_)	3.96	5.16	4.76
Threonine (C_4_H_9_NO_3_)	2.45	2.65	3.25
Cystine (C_6_H_12_N_2_O_4_S_2_)	0.25	0.05	0.00
Methionine (C_5_H_11_NO_2_S)	1.22	1.42	1.02
Leucine (C_6_H_13_NO_2_)	7.46	5.66	6.86
Isoleucine (HOCCH(NH)CH(CH)CHCH)	4.04	4.24	3.84
Lysine (C_6_H_14_N_2_O_2_)	3.49	3.69	3.29
Valine (C_5_H_11_NO_2_)	4.70	4.50	3.30
Tryptophan (C_11_H_12_N_2_O_2_)	0.20	0.00	0.07
Aspartic acid (C_4_H_7_NO_4_)	12.80	13.00	11.60
Glutamic acid (C_5_H_9_NO_4_)	21.00	22.80	20.20
Proline (C_5_H_9_NO_2_)	0.73	1.53	3.33
Serine (C_3_H_7_NO_3_)	4.25	5.45	3.05
Glycine (C_2_H_5_NO_2_)	2.56	3.36	4.76
Alanine (HOCCH(NH)CH)	3.65	2.85	4.45
Arginine (C_6_H_14_N_4_O_2_)	5.63	6.83	3.43
Histidine (C_6_H_9_N_3_O_2_)	1.79	2.99	0.59

**Table 3:** Antioxidant activities of methanolic crude fraction of *Vigna radiata* NM-92, NM-98, and NM-06 against DPPH radical.

Serial number	Meth. extracts	Concentration	Results	Standard n-propyl gallate
1	**NM-92**	500 *µ*g/mL	66.08%. ± 0.01	95.12% ± 0.01
2	**NM-98**	500 *µ*g/mL	64.15%. ± 0.01	
3	**NM-06 **	500 *µ*g/mL	62.11%. ± 0.01	

## References

[1] Adel A., Shehata Y., Thannoun A. M. (1980). Chemical and amino acid composition of Iraqi mung beans. *Zeitschrift für Lebensmittel-Untersuchung und Forschung*.

[2] Amarteifio J. O., Moholo D. (1998). The chemical composition of four legumes consumed in Botswana. *Journal of Food Composition and Analysis*.

[3] Anwar F., Latif S., Przybylski R., Sultana B., Ashraf M. (2007). *Chemical Composition and Antioxidant Activity of Seed of Different Culitivars of Mungbean*.

[4] Bhat R., Sridhar K. R., Seena S. (2008). Nutritional quality evaluation of velvet bean seeds (Mucuna pruriens) exposed to gamma irradiation. *International Journal of Food Sciences and Nutrition*.

[5] Bhatty N., Gilan A. H., Nagra S. (2000). Nutritional value of mung bean (*Vigna radiata*) as effected by cooking and supplementation. *Archivos Latinoamericanos de Nutrición*.

[6] Cavalieri A. K. J., Hunang A. H. C. (1980). Carrier protein-mediated transport of neutral amino acids into mung bean Mitochondria. *Plant Physiology*.

[7] Dzudie T., Hardy J. (1996). Physicochemical and functional properties of flours prepared from common beans and green mung Beans. *Journal of Agricultural and Food Chemistry*.

[8] Harper J. E., Corrigan K. A., Barbera A. C., Abd-Alla M. H. (1996). Hypernodulation of soybean, mung bean, and hyacinth bean is controlled by a common shoot signal. *Crop Science*.

[9] Wilson K. A., Chen J. C. (1983). Amino acid sequence of mung bean trypsin inhibitor and its modified forms appearing during germination. *Plant Physiology*.

[10] King R. D., Puwastien P. (1987). Effects of germination on the proximate composition and nutritional quality of winged bean (*Psophocarpus tetragonolobus*) seeds. *Journal of Food Science*.

[11] Thompson E. W., Laycock M. V., Ramshaw J. A., Boulter D. (1970). The amino acid sequence of Phaseolus aureua L. (mung-bean) cytochrome c.. *Biochemical Journal*.

[12] AOAC (1990). *Official Methods of Analysis*.

[13] Ullah R., Khader J. A., AbdEIslam N. M., Ullah F., Ullah M., Khan K., Ayaz S. (2013). Antioxidant activity of different crude fractions of *Sonchus eruca*. *Life Science Journal*.

[14] Agugo U. A., Onimawo I. A. (2009). Heat treatment on the nutritional value of mung bean. *Electronic Journal of Environmental, Agricultural and Food Chemistry*.

[15] Mubarak A. E. (2005). Nutritional composition and antinutritional factors of mung bean seeds (Phaseolus aureus) as affected by some home traditional processes. *Food Chemistry*.

[16] Bhatti M. I., Jamro G. H., Mangi S. U. (1990). Performance of wheat cultivar “Pavan” under different seeding rates and nitrogen levels. *Pakistan Journal of Agriculture, Agricultural Engineering*.

